# Growth and Replication of Infectious Bursal Disease Virus in the DF-1 Cell Line and Chicken Embryo Fibroblasts

**DOI:** 10.1155/2014/494835

**Published:** 2014-05-14

**Authors:** Kaliyaperumal Rekha, Chandran Sivasubramanian, Ill-Min Chung, Muthu Thiruvengadam

**Affiliations:** ^1^Department of Environmental and Herbal Science, Tamil University, Thanjavur, Tamil Nadu 613005, India; ^2^Department of Applied Bioscience, College of Life and Environmental Sciences, Konkuk University, Seoul 143701, Republic of Korea

## Abstract

Infectious bursal disease virus (IBDV) causes a highly contagious disease in young chicks and leads to significant economic losses in the poultry industry. To determine a suitable cell line for IBDV infection, replication, and growth kinetics of the virus, DF-1 cells and chicken embryo fibroblasts (CEF) were used. The population doubling per day (Pd/D) was found to be higher in DF-1 as compared to CEF cells. A suitable time of infection (TOI) was established for increased production of virus and greater infectivity titers. The DF-1 and CEF cells were found to be susceptible to infection by producing marked cytopathic effects (CPEs), and the growth curves of IBDV in DF-1 and CEF cells were evaluated by infectivity assay using tissue culture infectious dose (TCID_50_). The cytopathic effects of the virus in DF-1 and CEF cells were found to be similar, but higher viral titers were detected in the DF-1 cells as compared to CEF. Thus the DF-1 cell line had a higher growth potential and infectivity, which will be of advantage in vaccine production.

## 1. Introduction

Infectious bursal disease (IBD), also known as Gumboro disease, is caused by infectious bursal disease virus (IBDV). The virus causes a highly contagious disease in young chickens, with functional loss of the Bursa of Fabricius accompanied by severe immunosuppression [1], which leads to an increased susceptibility to other pathogens eventually resulting in greater mortality [2]. IBDV belongs to the genus* Avibirnavirus* of the Birnaviridae family and its viral genome is comprised of double stranded RNA [3]. Two serotypes of IBDV (1 and 2) were distinguished by cross-virus neutralization [4]. The IBDV strains of serotype 1 are pathogenic to chickens [5] and further classified as classical virulent IBDV (cvIBDV), very virulent IBDV (vvIBDV), antigenic variant IBDV (avIBDV), and attenuated IBDV (atIBDV) [6]. Strains of serotype 2 are naturally avirulent for chickens [7, 8]. As with all viruses, IBDV requires a receptor to penetrate target cells to cause infection. The distribution of this virus receptor mainly determines the target cells and the tissue specificity [9] and thereby the site of pathological changes associated with infection [10]. Chicken B lymphocytes are the primary target for virulent serotype 1 strains of IBDV, and the infection causes a functional loss of the Bursa of Fabricius and severe immunodepression. Recent advances in the understanding of the viral infection process have made it possible to develop new approaches to block the entry of viruses [11] and thus to prevent diseases [12]. However, a specific receptor on the surface of a susceptible host cell for the attachment of IBDV still needs to be identified.

Although virulent serotype strains of IBDV replicate efficiently in lymphoid cells of the Bursa of Fabricius in chickens, they are widely propagated in chicken embryo fibroblasts (CEF) [13]. But there are several disadvantages with the propagation of CEF cells. Their finite* in vitro* life span, high cost, and tedious, laborious preparation for continuous demand make it desirable to establish a new cell line of avian origin to replace CEF. DF-1 is an immortalized cell line of chicken embryo fibroblasts which has been demonstrated to support the growth of various avian viruses, including an avian sarcoma leukosis virus [14, 15], avian leukosis virus [16], Marek's disease virus [17], avian influenza virus [18, 19], and avian metapneumovirus [20, 21]. Another immortalized CEF cell line is SC-1 cells which, however, lack uniform cell morphology and do not exhibit a higher growth rate than DF-1 [22]. DF-1 cells arose spontaneously from line 0 (endogenous-virus negative) embryos [14] and do not harbor any known endogenous viruses [20]. Here we describe the growth kinetics of DF-1 and CEF cells, and the optimal time of infection (TOI) by IBDV and their susceptibility to infection were compared. A new effort has been made to study on the growth of DF-1 and CEF cell line and an estimated time of infection for enhancing increased virus production and infectivity titer were established. This approach would allow establishing an efficient cell line with increased virus yields that may find application in vaccine production against IBDV.

## 2. Materials and Methods

### 2.1. Cells

DF-1 cells were grown in 25 cm^2^ flasks with Dulbecco's modified Eagle medium (DMEM) (HyClone, USA) supplemented with 10% fetal calf serum (FCS) and 1% antibiotics (penicillin, streptomycin). The cells were passaged using Dulbecco's phosphate buffered saline (D-PBS), 0.25% trypsin (1X), and DMEM and maintained at 39°C in an incubator under an atmosphere of 5% CO_2_ (Sanyo, Japan). CEF cells were derived from specific-pathogen-free (SPF) 10-day-old chicken embryos and cultured in medium 199 (Gibco, USA) containing 10% FCS by standard procedures [23].

### 2.2. Virus Propagation

The locally isolated virulent IBDV strain R3 was adapted in chicken embryo fibroblast (CEF) cell culture and maintained at Rajiv Gandhi College of Veterinary and Animal Sciences, Pondicherry, India. Cells cultured in 25 cm^2^ flask were infected with 0.1 MOI (multiplicity of infection) of IBDV R3 and incubated at 39°C under 5% CO_2_ for 3 to 4 d. Cells were monitored every 24 h postinfection (h.p.i.) and inspected for cytopathic effects (CPEs) using an inverted microscope (Olympus CK 40, Japan).

### 2.3. Virus Harvesting

An infected monolayer was removed from the flask and transferred to Eppendorf tubes for further processing. The culture medium (viral suspension) was centrifuged at 1800 ×g for 10 min at 4°C to pellet cell debris. The clear supernatant was collected carefully, divided into aliquots, and stored at 4°C as viral stock for further use.

### 2.4. Analysis of Viral Growth in DF-1 and CEF Cells

Cell culture flasks (25 cm^2^) containing confluent monolayers of CEF or DF-1 cells were inoculated with 0.1 MOI of strain R3. After absorption for 2 h at 37°C, the inoculum was removed, and 5 mL of maintenance medium was added to each flask, which was then returned to the incubator. The viral suspension was collected at 24 h intervals postinfection and centrifuged. Activity assays were carried out with the supernatants in triplicate.

### 2.5. Infectivity Assay for Virus Yield

The monolayer of DF1 or CEF cells was washed with D-PBS two times and treated with 0.25% trypsin (1X) in DMEM. The cells were pelleted at 800 ×g for 10 min and resuspended in DMEM containing 10% FCS, then transferred to 96-well microtiter plates (Titretek, UK), and incubated for 1-2 h. The virus-containing supernatants were serially diluted (1 : 10) with growth medium. About 50 *μ*L of each dilution was transferred to the wells of a 96-well microtiter plate that contained the same volume of fresh DF-1 and CEF cells (3.0 × 10^5^ cells/mL) suspension medium. Cell suspensions without virus served as controls. Plates were sealed and incubated at 39°C in 5% CO_2_ atmosphere for 72–96 h. After staining with 1% crystal violet, cells were inspected under an inverted microscope for CPE, and virus titers were determined by the method of Reed and Muench [24].

### 2.6. Experimental Design and Data Analysis

All experiments were performed in triplicate and each experiment was repeated three times. The data were expressed as means ± standard deviation. One-way ANOVA analysis followed by Duncan's test was used to determine significant (*P* ≤ 0.05) differences. All the statistical analyses were done with the SPSS ver. 20 (SPSS Inc., Chicago, IL, USA) statistical software package.

## 3. Results and Discussion

**Figure 1:**
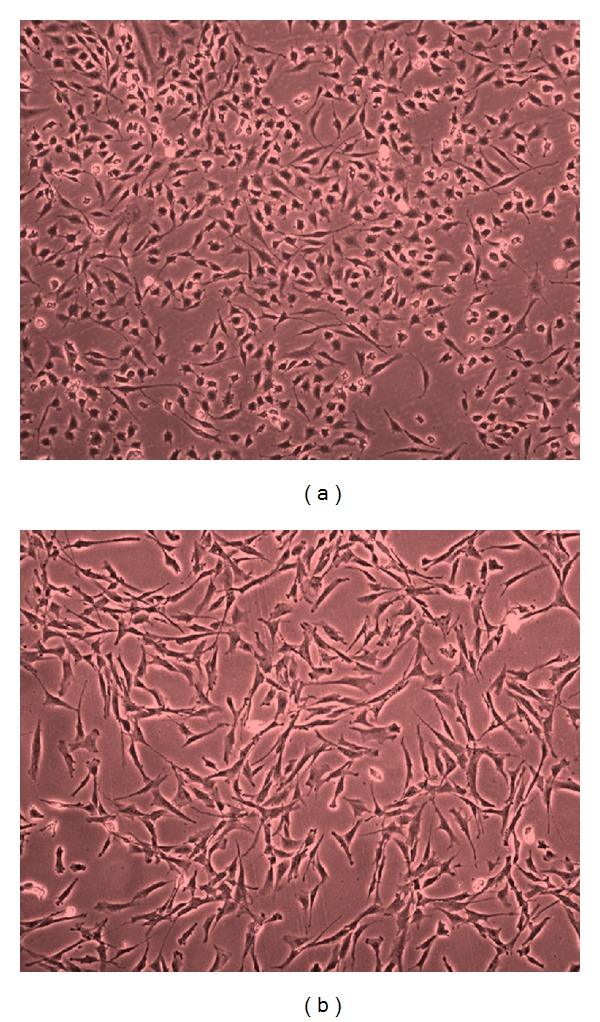
Normal cell morphology of DF-1 (a) and primary CEF (b) cells was observed by inverted microscopy at 100x magnification.

**Figure 2:**
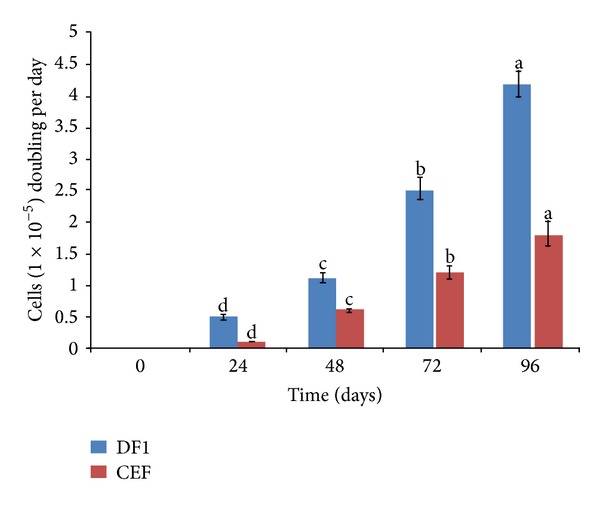
Growth kinetics of DF-1 and primary CEF. The cells were seeded at 1 × 10^5^ cells per 10 cm dish and total cell numbers were counted each day for 4 days. Data represent mean values ± SD of three replicates; each experiment was repeated three times. Means with common letters are not significantly different at *P* ≤ 0.05.

The DF-1 and primary CEF cells grew rapidly producing confluent monolayers, and their growth rates were determined during continuous passage. The DF-1 cells, even after prolonged passage, showed typical morphological characteristics of spindle-shaped fibroblasts but were clearly smaller in size, especially regarding cellular projections, compared to primary CEF cells (Figures 1(a) and 1(b)). DF-1 cells grew by 1.1 to 1.3 population doublings per day (PD/d) compared to 0.6–0.8 PD/d of primary CEF cells (Figure 2). Another spontaneously immortalized cell line, SC-1, has been reported to exhibit a lower growth rate ranging from 0.3 to 0.5 PD/d [22].

To propagate viruses in cell culture, a suitable time of infection (TOI) must be determined. Exponential growth of both DF-1 cells and CEF cells started after a lag phase of about 48 h, and cell concentrations were maximal at 1.29 × 10^6^ cells/mL at 72 h for DF-1 and at 1.0 × 10^6^ cells/mL at 96 h for CEF. After the respective maximum, cell concentrations declined and cells entered the death phase. Based on this, TOI was set between 72 h and 96 h when the cells were in the exponential phase. During this period, cells which could serve as virus replication hosts are highly available, and an optimal TOI will thus contribute to higher virus production.

**Figure 3:**
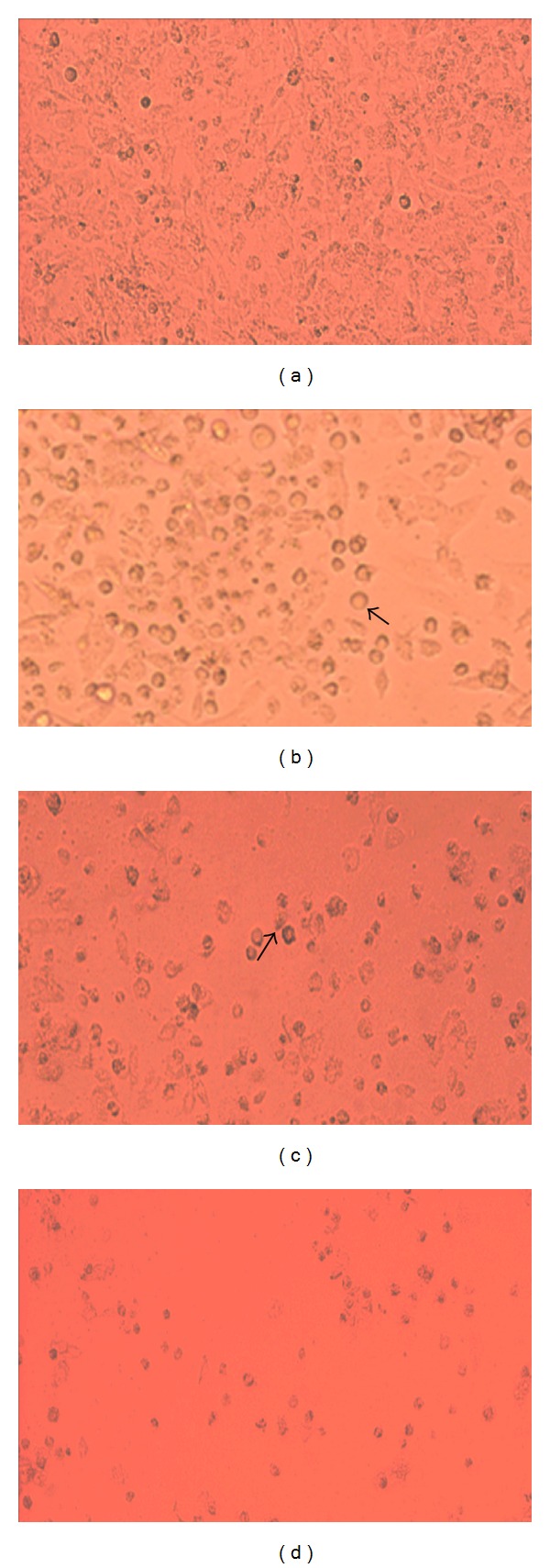
Cytopathic effects of infectious bursal disease virus (IBDV) in monolayers of DF-1 cells observed under an inverted microscope (100x) at various hours postinfection (h.p.i.). Monolayer at 24 h (a); granulation around nuclei (arrows) and cell rounding at 48 h (b) and 72 h (c); cell detachment from surface at 96 h.

The DF-1 and CEF cell lines were found to be susceptible to IBDV and exhibited similar cytopathic symptoms in response to viral infection. Several mammalian continuous cell lines have been reported to be susceptible to the IBDV infection, such as RK-13 cells derived from rabbit kidney [25], Vero cells derived from African green monkey kidney [26], BGM-70 derived from baby grivet monkey kidney [27], MA-104 derived from foetus rhesus monkey kidney [27], and OK derived from ovine kidney [28], and to support virus propagation with a distinct cytopathic effect (CPE) at low infectivity rate. In our experiments, IBDV infection produced a CPE characterized by a marked granulation of cell cytoplasm, particularly around the nucleus, and further resulted in cell rounding, followed by fragmentation of cells into small particles and finally detachment from the substrate, until eventually the entire monolayer was destructed (Figure 3). It has been reported that RK-13 cells replicated IBDV and exhibited CPE with virus titers similar to those of CEF cells [29]. Likewise, Vero cells were also reported to propagate this virus; however, initial passages do not produce visible CPE [30]. Therefore, finding an alternative cell line was crucial for propagating IBDV with increased titer. Moreover, DF-1 cells have been reported with increase viral titer, and it might be a stronger affinity of IBDV receptor on DF-1 cells, rather than the CEF cells [31]. Thus, the DF-1 cells may be useful for routine propagation of IBDV. However, all IBDV strains are not able to replicate in cell cultures, so the DF-1 cells may not be adequate for all IBDV vaccine strains.

**Figure 4:**
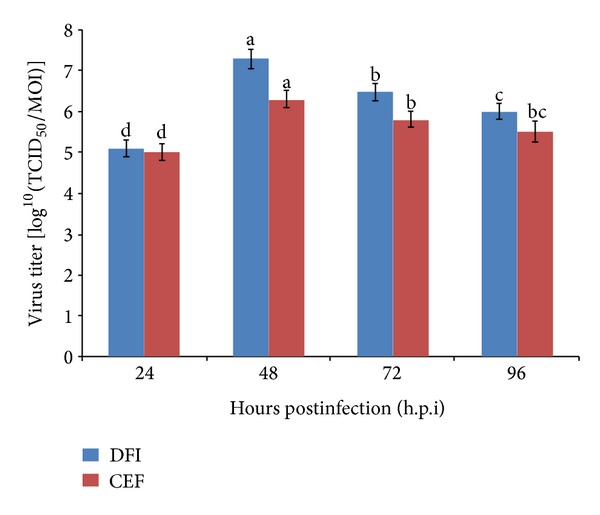
Analysis of virus titers in DF-1 and chicken embryo fibroblasts (CEF) cells at various hours postinfection using TCID_50._ Cell culture flasks (25 cm^2^) containing confluent monolayers of CEF or DF-1 cells were inoculated with 0.1 MOI of IBDV. Virus media were then harvested at indicated time points for titration using TCID_50_. The data represent the mean titer and standard deviation for each time point of the triplicate assays. Means with common letters are not significantly different at *P* ≤ 0.05.

**Figure 5:**
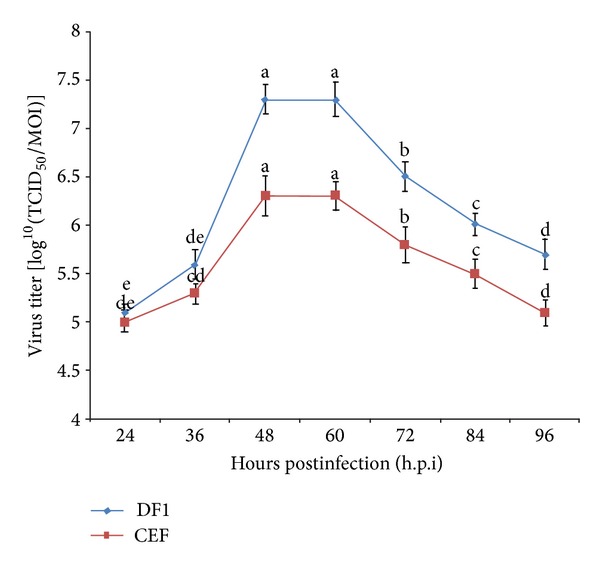
Growth curves of infectious bursal disease virus (IBDV) in DF-1 and chicken embryo fibroblasts (CEF) cells. The data represent the mean titer and standard deviation for each time point of the triplicate assays. Means with common letters are not significantly different at *P* ≤ 0.05.

To increase the production of virus and its titer, the DF-1 and CEF cell lines were infected in the exponential phase of their growth. Initially, virus growth determined every 24 h.p.i. (Figure 4). The growth curves of IBDV in CEF and DF-1 cells were found to be similar, but the titers varied. Further, to estimate its growth curve characteristics, the infectivity titer of IBDV was determined every 12 hours of postinfection (Figure 5). An observation of DF1 and CEF cells showed that until 12 h.p.i., the number of released viruses was similar for the two cell lines. Moreover, the lysis of host cells also showed no distinct difference between the two cell lines. The gradual changes in cell morphology were observed and CPE was noticed from 24 h.p.i. onwards (Figure 3(a)). The virus titers of CEF and DF-1 cells started to increase logarithmically at 24 h.p.i. At 48 h.p.i., the elongated cell morphology exhibited a marked granulation of cell cytoplasm, particularly around the nucleus, and cells appeared as rounded structures (Figure 3(b)) and a steady increase in CPE was observed. A plateau was reached at 48 h.p.i. and the virus titer increased more rapidly in DF-1 cells with a titer of approximately 7.3 while it was 6.3 (expressed in log^10^ TCID_50_) in CEF cells at the peak of virus production. In the later hours, from 60 h.p.i. onwards, the viral titer gradually decreased. At 72 h.p.i., the entire monolayer was destructed resulting in cell deterioration (Figure 3(c)), and detachment from the substrate was observed at 96 h.p.i. (Figure 3(d)). However, the viral titer in the DF-1 cells was still higher than in CEF cells at any time, confirming that DF-1 cells possessed an enhanced potential to produce virus compared to CEF cells.

PCR-based molecular diagnostic tools are more sensitive and efficient in virus diagnosis and quantification. However, it can be effective only with a known viral genome, and one may to fail to detect IBDV with noncharacterized or newly emerged genomes. Therefore, we have used the classic methodology to choose the best cell lines for IBDV propagation, and our results also support the previous studies by Wang et al. [31]. We hope our report may be useful for future studies to compare other cell lines and other virus replication studies.

## 4. Conclusion

The present study has been focused with the aim of better defining satisfactory and an efficient cell line for isolation and propagation of IBDV. When compared to primary chicken embryo fibroblast (CEF) cells, the DF-1 cell line exhibits enhanced growth rates. Based on its growth kinetics, the accurate time of infection (TOI) for increasing the production of virus and its infectivity titer were determined. Thereby, DF-1 cells infected also expressed an increased infectivity titer compared to CEF. Thus to conclude, if DF-1 cells in the exponential phase of growth were infected with infectious bursal disease virus could produce increased infectivity titer resulting in greater virus production. Besides various disadvantages in using CEF, this spontaneously immortalized nontransformed DF-1 cell line could provide an unlimited supply of identical cells resulting in increased production for vaccine development.
